# Hospital Questions Which Press

**Published:** 1905-02-18

**Authors:** 


### HOSPITAL ADMINISTRATION.

### CONSTRUCTION AND ECONOMICS.

### HOSPITAL QUESTIONS WHICH PRESS.

Want of Co-operation.

What evils are due at the present time to the absence of co-operation amongst the voluntary hospitals of London? Can they be remedied, and, if so, to what extent? These are questions of vital importance. At the present moment, when so much is being effected in different quarters to advance the welfare and increase the usefulness of the voluntary hospitals in London, these matters can be brought forward without any fear that they will be passed over without receiving that amount of consideration which they deserve. That there is a striking want of co-ordination in the hospital system as a whole probably no one who has interested himself in these matters will deny. In fact, it seems fairly well established that, not only do the individual voluntary hospitals neglect the advantages which would accrue to the public and to themselves as charitable institutions if they were to work hand-in-hand with their neighbours, but as educational centres, too, they lose considerably in value through this policy of isolation. For in this way a wide field for observation and experience is closed to those who are in process of learning their profession, and upon whose skill and attainments the public will in future time have to depend. Moreover, the absence of a proper unity of spirit and purpose is noticeable, not only amongst the voluntary hospitals themselves, but between them and the dispensaries, Poor-law infirmaries, private practitioners, and the various charitable institutions of London. Who is responsible for this deplorable state of affairs? Without doubt the managers of the voluntary hospitals themselves.

The more the question is studied from an impartial standpoint the greater does the conviction become that this want of co-operation is a very great evil, and the less difficult do the remedies for some of the drawbacks appear to be. There is, fortunately, good reason to anticipate that the managers of every general and special voluntary hospital in London will receive in a generous spirit any suggestions which may be offered towards a complete or partial solution of the difficulties and troubles which arise at the present time from this want of hospital co-operation.

Its Effects upon the Patients.

There are occasions when this want of free communication and mutual endeavour between the voluntary hospitals tells very hardly upon the patient who seeks, and is a proper subject for, admission into one of them. It is not necessary to illustrate this by any suppositious case; here is a real instance to hand taken from the life. A mother brought her child to the casualty room of one of the large general hospitals of London. The child was examined by the house physician on duty and found to be suffering from pneumonia, for which it was admitted as an in-patient. On inquiry it transpired that the mother had previously on the same day taken the child to no less than three other general hospitals, at each one of which she had been told that the child would have been admitted if there had been a vacant bed. As the child could not be taken in, the mother had been advised to take it on to the next general hospital and try there. In this way the wretched woman had wandered about London for a great part of the day carrying her infant with her, and waiting her turn in the various casualty rooms, until at last she had found a hospital which could give her the help she sought. It was not an instance of the hospital "cadger" so frequently met with in hospital casualty rooms; there was no doubt about the urgency of the case or its suitability for in-patient treatment. Why could not the house physician or casualty officer at the first hospital where the child was seen have kept the child and mother there, until it had been ascertained by telephone or other means at what hospital the child could be admitted? The answer is simple — because no arrangements whatever are made at the present moment by the managers of the hospitals for such co-operation among the different institutions which are under their charge. It was possible, no doubt, in the particular instance mentioned to have telephoned from the first hospital before the mother went away to discover which of the other hospitals had an empty cot to spare; but under the existing arrangements, or rather want of arrangement, this would be much too tedious and slow a process, and the members of the junior staffs of our hospitals have not sufficient time at their disposal to make a rule of adopting the practice generally. The case we have described is, of course, an extreme example, and such a one is probably rare; but it serves to illustrate in a striking manner a defect in hospital management which ought to be rectified without delay.

Another species of hospital malpraxis resulting from the present lack of intercourse between the voluntary hospitals is of considerable frequency. It occurs when a patient happens to apply for treatment at some hospital which is not fortunate enough to possess the proper means for affording him relief. Under these circumstances it is by no means the rule for the patient to be given the chance of going to another hospital which is supplied with the apparatus required for his particular case. Instead, he is usually subjected to such a course of treatment as can be carried out with the inferior appliances which are at the disposal of the hospital where he first attends. We could give instances of patients suffering from lupus who have been submitted to barbarous and inefficient applications of the actual cautery and caustics, because, forsooth, the hospital they chanced to select was not supplied with an electrical department, and could not therefore afford proper treatment for their maladies. This is indeed hospital malpraxis, and of a kind which it is difficult to extenuate. It is as unreasonable and senseless as it is pernicious, both to the welfare of the sick poor and the reputation of the voluntary hospitals themselves.

Evil Comparisons.

Another effect which this want of co-operation among hospitals exerts to the detriment of their usefulness among the poor is that which is exemplified by the manner in which a patient makes his selection of which hospital he will attend. He does not always go to the nearest hospital. He has learnt, as is natural under the present conditions, to look upon the different voluntary hospitals as rival institutions, and, to the best of his abilities, he is constantly making comparisons between them. The fact that a friend or relative of his once died in a certain hospital is likely to lead him to avoid that institution, wherever it may be, for the rest of his days; if, on the other hand, he has some acquaintance who imagines that his life was saved by the skill and care expended on him at another hospital, thenceforth that is the hospital for him, and he will go in consequence right across London, perhaps twice a week or more, to attend as an out-patient, and quite possibly to be treated when he gets there by the identical physician or surgeon under whose care he would be if he went instead to the hospital nearest to his own home. Not only is this a waste of labour and money, but there is pretty good evidence to show that if members of the working classes could be taught always to look first to their own local hospital for help in time of illness, they would very soon learn to take a much more lively pride and interest in that institution, and would accordingly take an increasing share in its support.

(*To be continued.*)

